# Brain Volumetric Correlates of Autism Spectrum Disorder Symptoms in Attention Deficit/Hyperactivity Disorder

**DOI:** 10.1371/journal.pone.0101130

**Published:** 2014-06-30

**Authors:** Laurence O’Dwyer, Colby Tanner, Eelco V. van Dongen, Corina U. Greven, Janita Bralten, Marcel P. Zwiers, Barbara Franke, Jaap Oosterlaan, Dirk Heslenfeld, Pieter Hoekstra, Catharina A. Hartman, Nanda Rommelse, Jan K. Buitelaar

**Affiliations:** 1 Radboud University Medical Center, Donders Institute for Brain, Cognition and Behaviour, Department of Cognitive Neuroscience, Nijmegen, The Netherlands; 2 Department of Ecology and Evolution, University of Lausanne, Lausanne, Switzerland; 3 King’s College London, Social, Genetic and Developmental Psychiatry Centre, Institute of Psychiatry, London, United Kingdom; 4 Department of Human Genetics, Radboud University Medical Center, Nijmegen, The Netherlands; 5 Radboud University Medical Center, Donders Institute for Brain, Cognition and Behaviour, Department of Psychiatry, Nijmegen, The Netherlands; 6 Department of Clinical Neuropsychology, Vrije Universiteit, Amsterdam, The Netherlands; 7 Department of Cognitive Psychology, Vrije Universiteit, Amsterdam, The Netherlands; 8 Department of Psychiatry, University Medical Center Groningen, University of Groningen, Groningen, The Netherlands; 9 Karakter Child and Adolescent Psychiatry University Center Nijmegen, Nijmegen, The Netherlands; Georgetown University, United States of America

## Abstract

Autism spectrum disorder (ASD) symptoms frequently occur in subjects with attention deficit/hyperactivity disorder (ADHD). While there is evidence that both ADHD and ASD have differential structural correlates, no study to date has investigated these structural correlates within a framework that robustly accounts for the phenotypic overlap between the two disorders. The presence of ASD symptoms was measured by the parent-reported Children’s Social and Behavioural Questionnaire (CSBQ) in ADHD subjects (n = 180), their unaffected siblings (n = 118) and healthy controls (n = 146). ADHD symptoms were assessed by a structured interview (K-SADS-PL) and the Conners’ ADHD questionnaires. Whole brain T1-weighted MPRAGE images were acquired and the structural MRI correlates of ASD symptom scores were analysed by modelling ASD symptom scores against white matter (WM) and grey matter (GM) volumes using mixed effects models which controlled for ADHD symptom levels. ASD symptoms were significantly elevated in ADHD subjects relative to both controls and unaffected siblings. ASD scores were predicted by the interaction between WM and GM volumes. Increasing ASD score was associated with greater GM volume. Equivocal results from previous structural studies in ADHD and ASD may be due to the fact that comorbidity has not been taken into account in studies to date. The current findings stress the need to account for issues of ASD comorbidity in ADHD.

## Introduction

Attention-deficit/hyperactivity disorder (ADHD) and autism spectrum disorder (ASD) are both severely impairing, highly heritable neurodevelopmental disorders [1], [2]. ASD is characterised by impaired social and communicative skills as well as restricted and repetitive behaviours and interests, whereas ADHD is characterised by severe inattention and/or hyperactivity and impulsivity [3]. Both disorders frequently co-occur with estimates for the presence of ADHD within ASD ranging from 30% to 80%, while the presence of ASD in ADHD is estimated at 20% to 50% [1], [2], [4]–[6]. Deficits in executive function and motor speed have been linked to familial vulnerability for both ASD and ADHD [7]–[9]. Studies have also documented an overlap of genetic factors that relate to both ASD and ADHD [4]. Overall, these findings suggest that there are shared etiological pathways for ASD and ADHD which need to be studied in more detail.

Quantitative measures that reflect the severity of ASD and ADHD symptoms may be useful to characterise both disorders in terms of a continuous distribution in which clinical disorders represent extreme variants of typical behaviour [10], [11]. Studies investigating the continuous distribution of both disorders as well as their overlap, as opposed to an exclusive focus on a categorical diagnosis, may be a fruitful direction for future research [10]. Such an approach may help to better characterise the heterogeneous nature of these disorders as well as the extent to which they overlap [12]. Previously, ASD symptoms have been found to be different in various groupings of ADHD classes, with the class having the most severe ADHD symptoms also having the most severe ASD symptoms [13]. In typically developing people, recent work has found brain regions that are commonly as well as uniquely correlated with ASD and ADHD symptom severity [14]. GM volume in the left inferior frontal gyrus was found to be correlated with symptom severity in both disorders [14]. Volumetric changes in the left posterior cingulate were found to be specific to ASD, while ADHD symptom severity was found to be specifically correlated with the right parietal lobe, right temporal frontal cortex, bilateral thalamus and left hippocampus/amygdala complex [14]. The study by Geurts et al. indicates that ASD and ADHD form a continuum extending into the general population. However, the conclusions were complicated by the fact that the directions of the brain-behaviour relationships were not consistent for all regions compared to clinical studies [14].

Brain volume abnormalities are important indicators of pathophysiological processes that likely reflect disorder etiology. A number of meta-analyses have found reduced brain volume in ADHD in the majority of studies investigated (with ages ranging from ∼10–37 years of age) [15], [16], [17]. Regional volume reductions in ADHD subjects were robustly localised to the globus pallidus, putamen, caudate nucleus and lentiform gyrus [15], [16], [17]. Total brain volume and grey matter volume have both been found to be decreased in ADHD compared with typically developing controls [15], [18] [19], [20]. However, both increasing age and stimulant medication were also found to be independently associated with more normal GM volumes.

It is of note that the profile of changes in brain volume in ASD is different from that of ADHD, with findings suggesting that the brain undergoes a period of accelerated growth in the first four years of life before reaching a plateau within a normal range by adolescence, which in turn is followed by volume decline in adulthood relative to typically developing controls [21], [22–27]. Importantly, in young children with autism (2–3 years old) WM enlargement has been shown to be greater than GM enlargement (18% more cerebral and 38% more cerebellar WM) [28]. This WM enlargement in young children was then found to be reversed in 12–16 year old children with autism, with WM volume reduced relative to controls [28]. In older individuals with autism, voxel-based methods have also shown less WM intensity than in age-matched controls [29,30]. However, a more recent meta-analysis has indicated consistent increases in WM volume in the right arcuate fasciculus and left inferior frontoocciptal and uncinate fasciculi in adults as well as in children and adolescents with ASD [31].

For GM, several studies in adolescents and adults with ASD have reported a 6–12% increase in GM volume relative to controls [32] [31], [33], [34]. A meta-analysis indicated a small increase in GM volume in the left middle and inferior frontal gyri in ASD [33]. The same meta-analysis also indicated robust reductions in GM in the amgydala-hippocampus complex and medical parietal regions [33]. Overall, the GM findings were found to be more pronounced in adults, but the only statistically significant difference was a greater GM volume reduction in the precuneus in adult compared with adolescent samples [33]. However it is difficult to disentangle whether these effects are due to developmental processes or are secondary to the effects of living with cognitive differences.

Significant age-related improvements in ASD have been documented in adolescents and adults [35]. This also suggests that a selection bias exists in current studies, with older participants possibly having more severe symptoms and thus more likely to have prominent GM abnormalities.

For ADHD, the ontogenetic onset of smaller brain size is not known, but there is some evidence that smaller brain volumes in ADHD persist to the end of adolescence [18]. However, this phenomenon may attenuate in adulthood, with studies noting normal brain size in adults with ADHD [36]. In summary, the most abnormal volumes in ASD generally occur in early childhood, while the most abnormal volumes in ADHD occur in middle to late childhood [15], [37], [38].

Therefore, while there is evidence that both ADHD and ASD have specific and differential structural correlates, no study to date has investigated these structural correlates within a framework that robustly accounts for the phenotypic overlap between the two disorders. Only one study to date has directly compared brain volumes in ASD and ADHD [39]. The age of all subjects ranged from 10 to 16 years, and no differences were found between the two disorders in terms of total GM and WM volumes [39] Additionally, no differences were found between either disorder and typically developing controls [39]. The relatively small size of the cohort (15 ASD subjects, 15 ADHD subjects, 15 controls) may have contributed to the negative findings.

The present study was based on the premise that the extent to which ASD symptoms occur in ADHD may have a pronounced effect on the profile of structural brain volumes in ADHD. Thus, MRI volumetric correlates of ASD symptoms in a large sample of ADHD probands, their unaffected siblings, as well as healthy controls were investigated. Mixed effects models, which controlled for ADHD symptom levels in the random effects structure of the model, were used to assess the relationship between global WM, GM and autism spectrum symptoms. As previous studies have found raised GM volume [32] in adolescent ASD subjects, we hypothesized that this profile would also be present in ADHD subjects with elevated autistic spectrum symptoms.

## Methods

### Participants

The study was approved by the Radboud University Medical Centre Research Ethics Committee and was in accordance with the Declaration of Helsinki. All participants over 18 years of age provided informed written consent. For participants under 18 years of age, informed written consent was obtained from the next of kin, caretakers, or guardians on behalf of the participants. This procedure was approved by the Radboud University Medical Centre Research Ethics Committee.

Participants were part of the NeuroIMAGE study. This is a prospective longitudinal MRI study (2009–2012) of the Dutch subsample of the International Multicenter ADHD Genetics (IMAGE) study performed between 2003–2006 [40]–[42]. All members of ADHD and control families were invited for follow-up measurement, with a mean follow-up period of 5.9 years (*SD* = .72). For the present analyses, subjects were selected from the total data set (n = 1084) when the following data was available: a high quality T1-weighted MPRAGE image, complete information from the Children’s Social and Behavioural Questionnaire (that provides information on the autism spectrum symptoms), complete information from the Schedule for Affective Disorders and Schizophrenia for School-Age Children - Present and Lifetime Version (K-SADS-PL) [43] and the Conners ADHD questionnaire. IQ information and medication history were also required in order to include subjects in the current study. Subjects with a sub-threshold ADHD diagnosis (n = 75), i.e. those with significantly elevated ADHD symptoms but failed to meet the clinical criteria for a diagnosis of ADHD were excluded. Subjects were also excluded if there was inconsistency between the ADHD diagnosis derived from the different questionnaires employed (n = 53). The resulting data set was then age- and gender-matched; so that the current study included 90 male and 90 female ADHD subjects, 62 male and 62 female unaffected siblings of ADHD subjects and 70 male and 70 female healthy control children. The age range of participants was 7.4 to 28.5 years. All participants were of European Caucasian descent and had an IQ≥70. Demographic characteristics of each subject group are provided in Table 1. Subjects who were taking ADHD medication stopped medication (both stimulant and non-stimulant) for 48 hours prior to scanning. Regarding medication history, 71 ADHD subjects (39%) were not currently taking ADHD medication, 93 ADHD subjects (52%) were taking one ADHD medication, and 16 ADHD subjects (9%) were currently taking two ADHD medications. A complete overview of medication is provided in Table S1.

**Table 1:** Demographic and cognitive characteristics of the sample groups.

	ADHD		Unaffected Siblings		Con		AVOVA’s	P	Tukey HSD		
	Mean	SD	Mean	SD	Mean	SD			US - Con	ADHD - Con	ADHD - US
Age (years)	16.2	3.7	16.9	4.0	16.8	3.6	F(2,441) = 1.41	0.25			
IQ	98.8	14.9	101.8	14.2	106.6	13.2	F(2,441) = 12.11	<0.0001	0.017	<0.0001	0.19
Social Interest (0–24)	4.3	4.3	1.9	3.4	1.0	1.9	F(2,441) = 40.42	<0.0001	0.12	<0.0001	<0.0001
Understanding (0–14)	5.5	3.7	1.2	1.5	1.2	1.8	F(2,441) = 140.1	<0.0001	0.99	<0.0001	<0.0001
Stereotyped (0–16)	2.1	2.4	0.4	1.0	0.4	1.0	F(2,441) = 50.3	<0.0001	0.99	<0.0001	<0.0001
Resistance to Change (0–6)	1.4	1.7	0.4	0.9	0.4	0.8	F(2,441) = 33.73	<0.0001	0.96	<0.0001	<0.0001
ASD-total (0–60)	13.3	8.9	3.9	5.0	3.0	4.3	F(2,441) = 118	<0.0001	0.54	<0.0001	<0.0001
ADHD-total	13.3	2.9	1.0	1.5	0.4	0.9	F(2,441) = 2008	<0.0001	0.089	<0.0001	<0.0001
Handedness (r/l/a)	152/24/3		101/17/0		126/19/1						

Values are mean ± standard deviation. Significance was set at p<0.05. All p-values refer to ANOVAs, except for gender where the p-value refers to a chi-square test. Where ANOVA’s returned a significant result, post-hoc Tukey Honest Significant Difference (Tukey HSD) tests were performed. US - Con, refers to a pairwise comparison between unaffected siblings and controls. ADHD-Con, refers to a a pairwise comparison between ADHD and controls. ADHD - US, refers to a pairwise comparison between ADHD and unaffected siblings. Four subscales of the Children’s Social and Behavioural Questionnaire (CSBQ) which probe ASD spectrum symptoms are shown in this table: Social Interest, Social Understanding (Understanding), Stereotypy and Resistance to Change. ASD-total is calculated as a sum of these four subscales. ADHD-total scores are calculated according to the algorithm described in detail in the supplementary information (Questionnaire Items S1).

Abbreviations: Gender (m/f), Gender (male/female); Handedness (r/l/a), Handedness (right, left, ambidextrous).

### ADHD diagnosis and symptom measures

All participants were assessed using a semi-structured diagnostic interview (Dutch translation of the Schedule for Affective Disorders and Schizophrenia for School-Age Children - Present and Lifetime Version (K-SADS-PL) [43] and Conners’ ADHD questionnaire to determine ADHD diagnoses. Each child was assessed with a parent-rated questionnaire (Conners’ Parent Rating Scale – Revised: Long version (CPRS-R:L) combined with either a teacher-rating (Conners’ Teacher Rating Scale – Revised: Long version (CTRS-R:L) applied for children <18 years or a self-report Conners’ Adult ADHD Rating Scales – Self-Report: Long version (CAARS-S:L) applied for children ≥18 years. A diagnostic algorithm was applied to combine symptom counts on the K-SADS-PL and Conners’ questionnaires. A detailed description of the diagnostic algorithm is provided in the supplementary information (Diagnostic Algorithm S1). This algorithm was used to diagnose subjects and also to provide a continuous score for an overall level of ADHD symptoms that combines both inattentive and hyperactive symptoms. While ASD symptoms were present in the cohort and were significantly elevated in the ADHD group (see Results), a clinical diagnosis of autism or Asperger’s syndrome had been an exclusion criterion in the original IMAGE study.

### ASD measures

The Children’s Social and Behavioural Questionnaire (CSBQ) [44] was used in the current study as it can measure the continuous distribution of autistic symptoms in the general population as well as in clinical groups outside of autism, including ADHD [44], [45]. Most instruments for ASD are designed to assess clinical cases and are not suitable for assessing milder ASD symptoms. The CSBQ was originally developed because existing instruments did not capture more subtle social problems found in the milder end of the autism spectrum [44]. The CSBQ has previously been shown to be valuable in measuring subthreshold autistic symptoms [44]–[49], [40]. In the current study, where clinical ASD was an exclusion criterion, but where milder autistic symptoms are expected to be elevated in the ADHD group [2], [4]–[6], [50], the CSBQ is ideally suited for the purpose of the study.

The CSBQ contains 49 items on a 3-point Likert scale. It contains items that refer directly to the DSM-IV criteria for autism, but it also captures more subtle symptoms of ASD. Therefore, it is suitable for measuring behavioural problems as seen in children with milder variants of ASD. CSBQ items are grouped into the following six subscales: (1) tuned, (2) social interest, (3) orientation, (4) social understanding, (5) resistance to change, and (6) stereotypy. It was administered in the parent-report form.

All items contained within each subscale are found in the supplemental information (Questionnaire Items S1). The CSBQ has good internal, test-retest, and inter-rater reliability, and demonstrated convergent and divergent validity [44]. The CSBQ appears to differentiate between autism and pervasive developmental disorders - not otherwise specified (PDD-NOS), and can also differentiate between PDD-NOS and ADHD [44], [51]. Additionally, to assess the content validity of the CSBQ, it has previously been compared to an autism screening instrument, the Autism Behavior Checklist (ABC) [52]. A strong correlation of 0.75 was found between the total scores of both questionnaires in a large Dutch population sample [45]. The CSBQ has also been compared with the Autism Diagnostic Interview-Revised (ADI-R), Autism Diagnostic Observation Schedule (ADOS), and clinical classification in children with mild and moderate intellectual disability [53]. In that study, the contribution of the CSBQ to a classification of ASD was most specific for the “social” and “stereotypy” subscales, with high coherence with all three classification methods [53].

An aggregate score from the four subscales, (1) social interest, (2) social understanding, (3) stereotypy and (4) resistance to change, was used in the current study to capture the core symptoms of ASD. This aggregate score has been successfully used to selectively probe ASD symptoms in previous studies [47], [54]. The remaining two CSBQ subscales (tuned and orientation) probe dysfunctional social behaviours which, although characteristic for ASD, are also related to the ADHD dimensions of hyperactivity/impulsivity and attention problems, respectively [44], and were not considered in the current study for this reason.

### High Resolution T1W Structural Image Acquisition and Processing

Whole brain T1 weighted MPRAGE images were acquired at 1.5T using a product 8 channel phased array headcoil on a Siemens Sonata scanner at the Free University in Amsterdam and a 1.5T Siemens Avanto MR scanner at the Donders Institute for Brain, Cognition and Behaviour in Nijmegen. A breakdown of the distribution of subjects scanned at the two sites is included in Table S2. TI/TE/TR = 1000/2.95/2730 ms, imaging matrix 256×256, 176 slices, voxel size 1×1×1 mm^3^, GRAPPA acceleration 2. Brain tissue probability maps for white matter (WM), grey matter (GM) and cerebrospinal fluid (CSF) were estimated using the unified segmentation algorithm as implemented in the VBM8 toolbox of SPM8 [55]. Total GM, WM and CSF volumes were computed by voxelwise summation of their probability maps. Total intracranial volume (ICV) was then computed as the sum of WM, GM and CSF volume.

The following formula was used to compute normalised volumes of global GM and WM:

total volume of GM (mm^3^)/ICV (mm^3^)×1000.
total volume of WM (mm^3^)/ICV (mm^3^)×1000.

### Statistics

As ASD scores are influenced by a range of factors, mixed-effects models allow us to consider factors that potentially contribute to the understanding of the data (i.e. fixed factors), while controlling for additional factors associated with the subjects (i.e. random factors).

R statistical software (Version 2.15.1) [56], including the lme4 package [57] was used for all statistical analyses. In the current study, the “glmer” function [57] was used to fit a generalised mixed-effects model using maximum likelihood (ML). As our objective was to determine the extent to which the ASD score is influenced by the diagnosis group (i.e. control, unaffected sibling or ADHD), GM and WM, these three factors were set as fixed effects. Age was also set as a fixed factor. The total ADHD symptom score was set as a random effect, in order to control for differences in ADHD symptoms between participants, thus allowing us to focus specifically on the ASD score. IQ, ADHD medication, scanner type and family relatedness were also set as random effects. The response variable was set as ASD score, calculated as an aggregate of the four CSBQ subscales (‘social interest’, ‘social understanding’, ‘stereotypy’ and ‘resistance to change’) that probe ASD.

Models were created using normalised WM and GM volumes. Including both WM and GM variables allowed us to determine not only the extent to which GM and WM drive changes in ASD scores, but also the nature of the interactions between the explanatory variables. WM and GM volumes were co-linear (i.e. correlated with each other) and the models developed took this into account by orthogonalizing these regressors relative to each other to isolate the unique contribution of each explanatory variable independent from what is shared between them. A WM residual term was derived from the regression residuals when WM was regressed against GM. This enabled us to use GM together with a WM residual term (WM resid) in our mixed effect model. A model using WM together with a GM residual term (GM resid) was also created. Un-biased analyses and comparisons could then be carried out based on the models.

The models were fitted by maximum likelihood estimation. The starting model using GM resid and WM terms was:
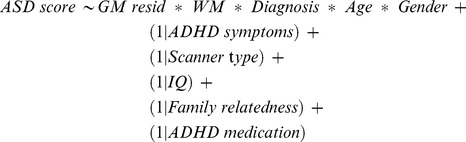
where “∼” means “modelled against”; “(1| factor)” means that factor is included as a random effect, and “Diagnosis” refers to the three diagnostic groups, i.e. control, unaffected siblings and ADHD. “*” means interacting with, and the model assesses interactions among all terms, up to a 5-way interaction among all fixed effects.

The starting model using GM and WM resid terms was:
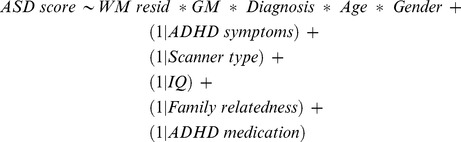
We fit the full models as described above and then removed least significant terms in an iterative process, checking for improved fit according to Akaike’s Information Criterion (AIC) [58], [59] until a final model was obtained [60]. We have previously employed the AIC tool for successful model selection in an MRI and structural volume framework [61]. The models are thus primarily assessing the influence of GM and WM on the ASD scores across all healthy young controls, ADHD subjects and unaffected siblings (Sib) of ADHD subjects.

To determine if the model using GM resid or the WM resid term was a better predictor of ASD symptoms, the fits of the two final models was compared using the “anova” function in R [60].

### Contour Plots

The significant interaction between GM and WM was visualized by means of a contour plot. Normalised WM and GM volumes were used for creating this contour plot. ASD scores were modelled as follows: model = lm (ASD score∼GM*WM resid); where “∼” means modelled against, “*” means interacting with, and, “lm” is used to fit a linear model in R. A matrix was created for the WM and GM volumes, and the function was run from the minimum to the maximum GM value within the matrix, and also from the minimum to the maximum WM value within the matrix. The contour plot enabled changes in ASD scores to be visualised by means of a colour key with darker colours representing lower ASD symptoms and lighter colours representing higher ASD symptoms.

## Results

### Demographic and Cognitive Characteristics

**Figure 1:**
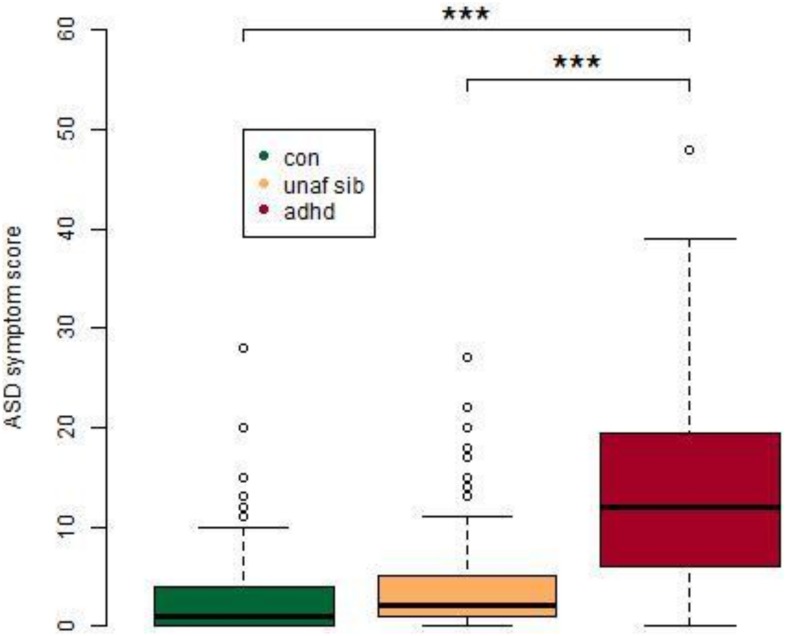
ASD symptoms in healthy controls, unaffected siblings and ADHD. ADHD subjects were found to have significantly higher scores relative to both unaffected siblings and healthy controls. ***p<0.001, with post-hoc Tukey test, following an ANOVA. ASD symptom score refers to an aggregate score from the four Children’s Social and Behavioural Questionnaire (CSBQ) subscales, (1) social interest, (2) social understanding, (3) stereotypy and (4) resistance to change. Abbreviations: con, Control; unaf sib, Unaffected Siblings, adhd, Attention Deficit Hyperactivity Disorder.

The demographic characteristics of the cohort are shown in Table 1. ASD scores were significantly different between groups [F(2,441) = 118, p<0.0001], with ADHD subjects having significantly raised scores relative to both controls (p<0.0001) and unaffected siblings (p<0.0001), while there was no significant difference between controls and unaffected siblings (Fig. 1; Table 1). ASD scores were found to be significantly positively correlated with ADHD scores in control group (p = 0.046, r = 0.165; Table S3) and in the ADHD group (p = 0.254, r = 0.001; Table S3).

### Mixed-effects models for ASD symptoms modelled against global grey matter and white matter volumes

Following model simplification based on AIC, the optimal model for ASD symptoms is shown in Table 2 and Table 3. This model used the WM residual term together with GM (WM resid:GM model) and was found to have a lower AIC value than the alternative model that used the GM residual term together with the WM (GM resid:WM model). The alternative model is also shown in Table S4.

**Table 2:** Final, generalised mixed-effect model showing fixed effects for ASD score modelled against white matter residual and grey matter volumes.

Fixed Effects	Estimate	Standard Error	t-value	p-value
WM resid	−0.485	0.206	−2.354	0.019
GM	0.013	0.013	0.994	0.321
Unaff. Sib.	−0.538	3.652	−0.147	0.883
ADHD	21.056	3.815	5.519	0.000
Age	0.071	0.177	0.398	0.691
Male	1.847	0.674	2.740	0.006
WM resid×GM	0.001	0.000	2.199	0.028
Unaff. Sib×Age	0.059	0.211	0.279	0.781
ADHD×Age	−0.675	0.207	−3.264	0.001

Formula: 
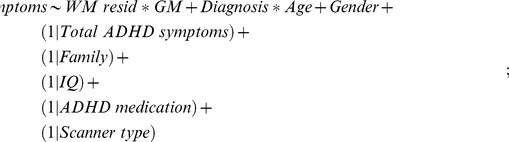

“∼” means modelled against, and “(1| factor)” means that a factor is included as a random effect.

A generalised mixed-effect model is run using normalised volumes of grey and white matter as explanatory variables together with age as a random effect. ASD score is set as the response variable. The final model is derived following an iterative model selection procedure that involves comparing successive models using Akaike’s Information Criterion (see Methods for detailed description of model selection procedure). ASD score refers to an aggregate score from the four Children’s Social and Behavioural Questionnaire (CSBQ) subscales, (1) social interest, (2) social understanding, (3) stereotypy and (4) resistance to change.

Abbreviations: WM, normalised WM volume; GM, normalised GM volume; US, unaffected siblings; WM:GM, WM by GM interaction; US: Age, unaffected sibling by Age interaction; ADHD:Age, ADHD by Age interaction.

**Table 3:** Final, generalised mixed-effect model showing random effects for ASD score modelled against white matter residual and grey matter volumes.

Random Effects	Variance	S.D.
Family Relatedness	8.765	2.961
IQ	1.098	1.048
Total ADHD Score	5.474	2.340
ADHD medication	0.000	0.000
Scanner Type	0.000	0.000

Formula: 
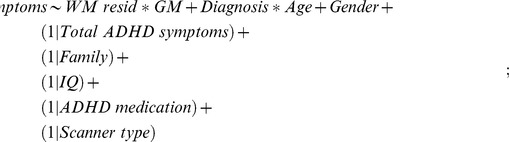

“∼” means modelled against, and “(1| factor)” means that a factor is included as a random effect.

A generalised mixed-effect model is run using normalised volumes of grey and white matter as explanatory variables together with age as a random effect. ASD score is set as the response variable. The final model is derived following an iterative model selection procedure that involves comparing successive models using Akaike’s Information Criterion (see Methods for detailed description of model selection procedure). ASD score refers to an aggregate score from the four Children’s Social and Behavioural Questionnaire (CSBQ) subscales, (1) social interest, (2) social understanding, (3) stereotypy and (4) resistance to change.

Abbreviations: WM, normalised WM volume; GM, normalised GM volume; US, unaffected siblings; WM:GM, WM by GM interaction; US: Age, unaffected sibling by Age interaction; ADHD:Age, ADHD by Age interaction.

For the fixed effects of the WM resid:GM model, the following terms were significant: WM resid (p = 0.019); ADHD diagnosis (p<0.0001); male gender (p = 0.006); WM resid×GM interaction (p = 0.028); ADHD diagnosis×age (p = 0.001).

As the range of ASD scores in control and unaffected siblings is limited, a mixed effect model was also developed based on the inclusion of participants with an ASD score of 20 or higher. This model also returned a significant WM resid×GM interaction (p = 0.0029; Table S5).

**Figure 2:**
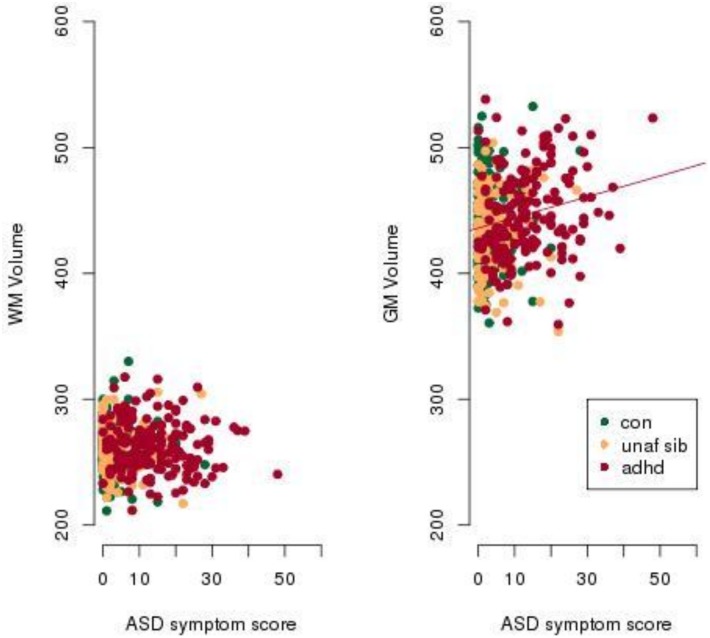
ASD scores are significantly positively correlated with total grey matter volume. The volumes of grey and white matter are normalised for total intracranial volume. A regression line is plotted where the Pearson’s product-moment correlation is significant with p<0.05. ASD symptom score refers to an aggregate score from the four Children’s Social and Behavioural Questionnaire (CSBQ) subscales, (1) social interest, (2) social understanding, (3) stereotypy and (4) resistance to change. Abbreviations: con, Control; unaf sib, Unaffected Siblings, adhd, Attention Deficit Hyperactivity Disorder; ASD, autism spectrum disorder.

**Figure 3:**
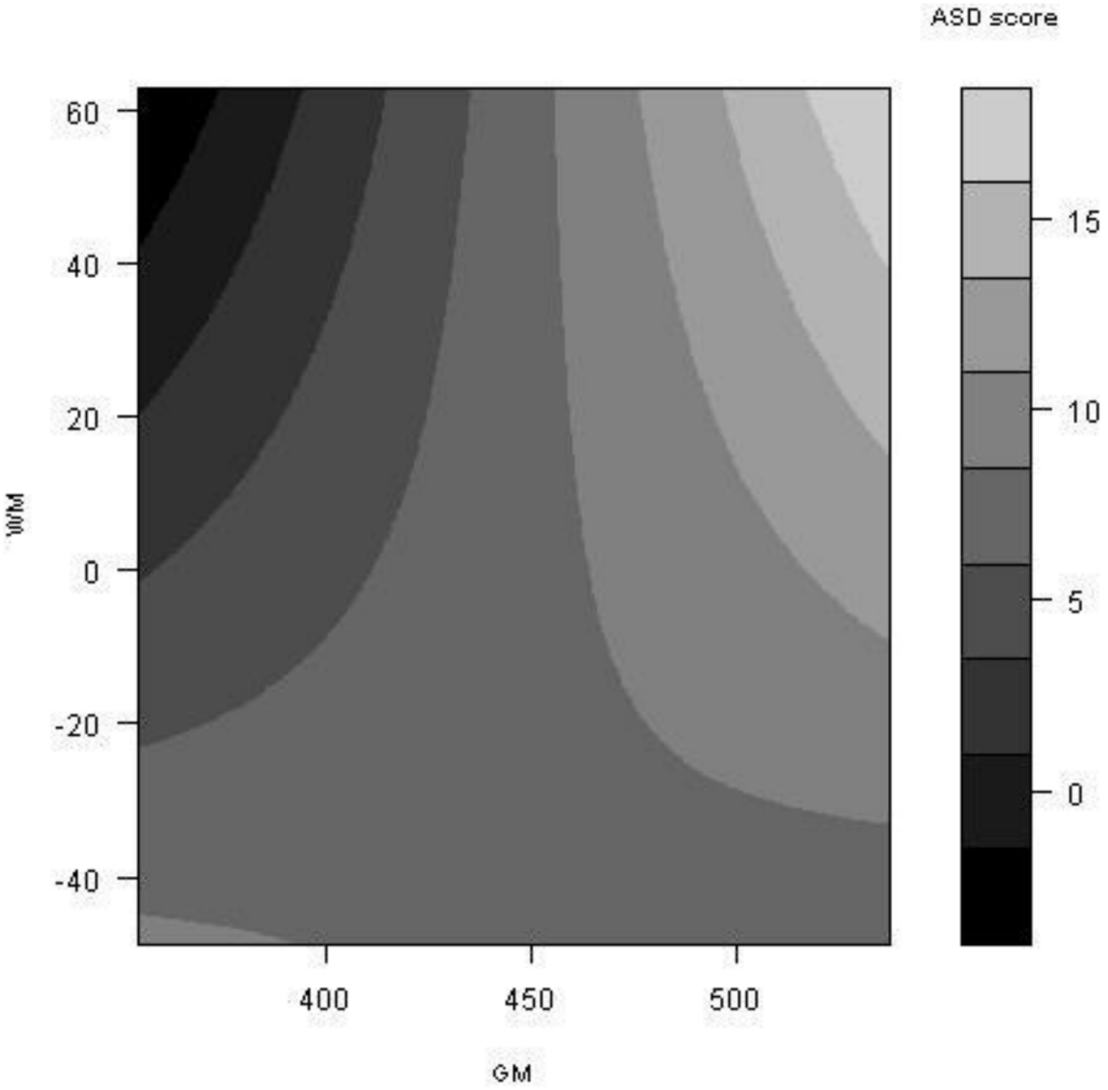
ASD score contour graph for the interaction between WM, GM and ASD scores. The best mixed effects model was converted into a function in R which allowed ASD scores to be extrapolated for a range of GM and WM volumes (see Methods for full description). The best mixed effects model included GM and WM residual terms (see Methods). Volumes are normalised for total intracranial volume. ASD score refers to an aggregate score from the four Children’s Social and Behavioural Questionnaire (CSBQ) subscales, (1) social interest, (2) social understanding, (3) stereotypy and (4) resistance to change.

The significant interaction between WM and GM was further visualised with a scatter plot (Fig. 2) and a contour plot (Fig. 3). A significant positive correlation was found between GM volume and ASD scores for the ADHD group (p = 0.00025, r = 0.17) (Fig. 2). No other significant correlation was found between GM volume and ASD score for control or unaffected siblings. There was also no significant correlation between WM volume and ASD score for any of the groups examined. The best mixed effects model (Table 2 and Table 3) was used to create a contour plot, which further described the interaction between GM and WM. The contour plot developed from the mixed effects model indicates that the lowest ASD scores were found in areas of low GM coupled with high WM (Fig. 3). Increasing ASD score was accompanied by greater GM volume and lower WM volume up to an ASD score on the CSBQ of ∼10. For ASD scores greater than ∼10, the volumetric profile indicated both raised GM and WM volume (Fig. 3).

**Figure 4:**
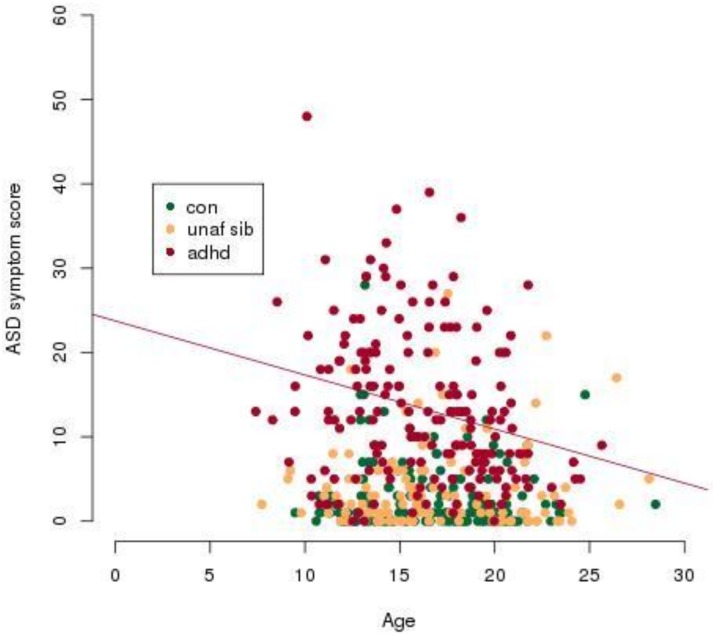
ASD scores decrease significantly with age within the ADHD groups. A regression line is plotted where the Pearson’s product-moment correlation is significant with p<0.05. ASD score refers to an aggregate score from the four Children’s Social and Behavioural Questionnaire (CSBQ) subscales, (1) social interest, (2) social understanding, (3) stereotypy and (4) resistance to change. Abbreviations: con, Control; unaf sib, Unaffected Siblings, ADHD, Attention-deficit/hyperactivity disorder; ASD, autism spectrum disorder.

As there was a significant interaction found between the ADHD diagnosis and age, this interaction was also visualised with a scatter plot (Fig. 4). ADHD subjects were found to have a significant negative correlation between age and ASD scores (p<0.0005; r = −0.26) while there was no correlation between age and ASD scores for unaffected siblings or controls (Fig. 4).

### Total Intracranial Volume in Low and High ASD Conditions

**Figure 5:**
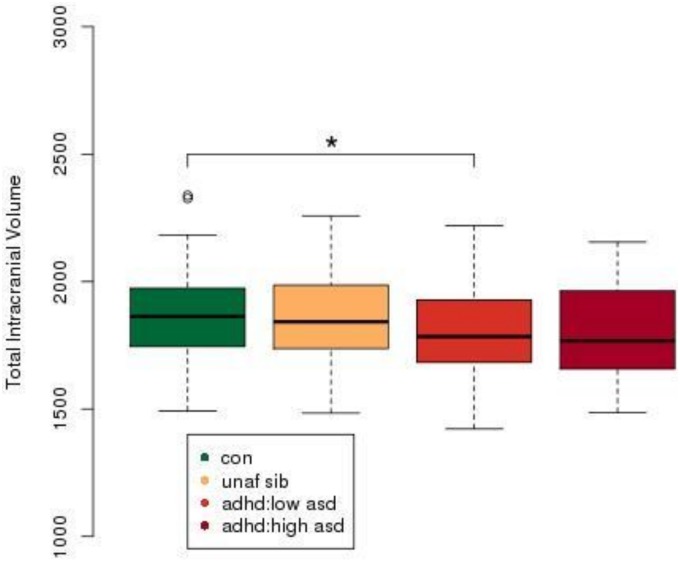
ADHD subjects with low ASD scores have significantly lower total intracranial volume than control subjects (p<0.05). ADHD: low ASD is defined as a participant with ADHD and an ASD score of 20 or less. ADHD: high ASD is defined as a participant with ADHD and an ASD score greater than 20.

Total intracranial volume was plotted for Control, Unaffected Sibling, ADHD with low ASD and ADHD with high ASD scores (Fig. 5). Low ASD score was defined as 20 or lower as per the CSBQ ASD score. A high ASD score was defined as greater than 20. ADHD participants with low ASD scores were found to have significantly lower total intracranial volumes than control subjects (p<0.05). No other significant differences were found.

## Discussion

In the current study, ASD symptoms as measured by the CSBQ [44] were found to be significantly elevated in ADHD subjects relative to both controls and unaffected siblings. These findings agree with previous studies that have found elevated levels of ASD symptoms in ADHD subjects [13], [62]. There was a non-significant increase in the ASD scores for unaffected siblings relative to controls, which is in line with studies showing moderate increases in ASD symptoms in unaffected siblings of ADHD subjects [40], [63]. For ADHD subjects, the co-morbid ASD symptoms were found to decrease significantly with age.

Structural MRI was used to investigate whether or not raised ASD symptoms would be accompanied by a distinct anatomical profile. When ASD scores were modelled against WM and GM volumes using mixed-effects models that controlled for ADHD symptom levels, a significant WM by GM interaction was found. Investigation of the interaction between WM, GM and ASD scores by means of a contour graph highlighted a specific volumetric landscape with the lowest ASD scores found in areas of low GM coupled with high WM. Increasing ASD scores were accompanied by greater GM volumes and lower WM volumes. While the GM profile constantly increased with greater ASD scores, WM was found to decrease with greater ASD scores but for the highest ASD scores WM volumes were also elevated.

The profile of increased GM volume in subjects with raised ASD symptoms is in line with previous studies that have found GM volume to be significantly increased in adolescent ASD subjects [32]. Globally, decreased brain size has been the predominant finding in ADHD [15], [18], while ASD is generally associated with brain overgrowth during infancy followed by a return to normal total brain size in adolescence and possible continued degeneration into middle age [64] [22]–[27]. Similarly, although the majority of studies have found total brain volume to be reduced in ADHD, some studies have reported no significant differences in total brain volume between ADHD subjects and typically developing controls [36], [65]–[68]. These discrepancies may be partly accounted for by the extent to which ASD symptoms are present in the ADHD sample. The findings in the current paper that ADHD with low ASD score had significantly reduced total intracranial volume compared with control, whereas ADHD with high ASD score showed no significant difference with control, further emphasize the role that ASD symptoms may play in influencing the landscape of brain volumetrics in ADHD subjects.

Meta-analyses suggest that increasing age and medication treatment contribute to a degree of normalisation in brain volumes in ADHD [16], [17]. The current findings add to these studies by indicating that ASD comorbidity may be an additional factor that plays an important role in influencing brain volume in ADHD. Decreased GM volume may be more pronounced when ASD symptoms are absent or present only to a minor degree, as was the case in the current study. Conversely, the current findings indicate that elevated ASD symptoms are accompanied by raised GM volume. The WM profile was more complex, with lower volumes found as ASD scores increase, but higher volumes found for the most extreme ASD scores. Previous studies have shown reduced WM volume in adolescent and adult ASD subjects [28], [29], [30], while greater WM enlargement has been found in children with ASD [28]. A mixed effects model assessment, based on the same statistical framework as the main analysis, but including only participants with an ASD score of 20 or higher also revealed a significant GM by WM interaction. This supplementary analysis indicates that those with elevated ASD scores in the current cohort have a global volumetric profile similar to that found in ASD studies.

The volumetric landscape for different ASD scores described by the contour plot is not restricted to ADHD subjects but is also applicable to unaffected siblings and healthy controls. No significant interaction between GM, WM and ADHD was found, nor were there significant interactions between GM, WM and unaffected siblings, or between GM, WM and controls.

As ASD scores remained low in both controls and unaffected siblings, their volumetric profiles were constrained to a conjunction of low GM coupled with high WM volume. Conversely, ASD scores for ADHD subjects were more heterogeneous, with a significantly higher mean, as well as greater variance and range. Therefore, ADHD subjects exhibited a wide range of volumetric profiles that were dependent on their ASD spectrum scores.

There was a significant age-related improvement in ASD symptoms in ADHD subjects which is in agreement with previous cross-sectional and longitudinal studies that have also found age-related improvement in ASD [69], [70] [71]. Neither WM nor GM volume was found to influence this age-related reduction of ASD symptoms in ADHD subjects. Therefore, the neural correlates of this finding remain unknown. Analyses of regional brain volume changes, as well as changes in WM indices of diffusion will be undertaken in a future study and may be able to shed light on this point. One possibility is that while total WM or GM volume may not have an influence on age-related changes in ASD symptoms, subtle regional changes in GM and WM structure changes may lie hidden beneath the global analysis. Structural and functional MRI studies have consistently found the caudate nucleus to be altered in ASD and to be associated with dysfunctions in multiple domains related to ASD, such as repetitive and stereotyped behaviour [72], reward processing [73]–[75] and executive function [73]. Caudate damage has also been related to reduced cognitive flexibility and perseverative behaviour in a range of species, including monkeys [76], birds [77], rats [78] and cats [79]. Clearly, developmental changes play a crucial role in influencing the trajectories of both disorders. Significant age-related improvements have been noted in restricted and repetitive behaviours [80] and also in overall ASD symptom counts [81]. The significant reduction of ASD scores may be related to a reduction of local short-range circuitry together with a strengthening of long-range connectivity that is seen in adolescent brain development [82]. Normal development is associated with marked changes in WM tracts, with myelination increasing throughout childhood and adolescence [83]. Increasing myelination of fronto-striatal connections during adolescence and early adulthood facilitates top-down executive control of behaviour [84]. Indices of WM integrity such as fractional anisotropy have been found to normalise in ADHD subjects as they progress to adolescence and adulthood [85]. Thus, the apparent improvements in autism symptoms with age as seen in the current study may be associated with improvements in WM integrity. Social adaptation may contribute to a reduction of ASD symptoms with age. Initial studies have also indicated that cognitive behavioural therapy (CBT), when adapted to the special needs of patients with ASD, can offer an effective treatment modality [86], [87], [88]. Although there are no pharmacological treatments that specifically target the deficits of ASD, some studies have noted improvement in behavioural symptoms with risperidone [89], while reductions in repetitive behaviours have been found following treatment with oxytocin [90]. Within the current cohort, individuals with low IQ (<70) were excluded, thus an IQ within a normal range may also facilitate compensation or adaptive mechanisms that help ADHD individuals to reduce the overt expression of ASD symptoms.

The current study should be viewed in the context of some limitations. The results are cross-sectional. Future longitudinal studies of MRI-measured developmental trajectories are needed for assessing the impact of age on developing brain structures. The results are also based on global volumetric changes and do not identify specific regional increases or decreases in WM or GM volume. Studies are underway which will assess the current data set in terms of specific regional WM and GM volumetric differences that occur with elevated ASD symptoms. The fact that clinical ASD cases were not included in the cohort is an additional limitation of the study. While the ASD score derived from the aggregate of four sub-scales of the CSBQ questionnaire (social interest, social understanding, stereotypy and resistance to change) has been successfully used to probe ASD symptoms in the past [47], [54], caution is needed when interpreting this score as not all social deficits present in ADHD overlap with ASD symptoms [91]. The fact that participants were of European (Caucasian) descent is another limitation, and indicates that results may not be readily generalisable to other communities or ethnicities.

Overall, the current results highlight a specific volumetric profile that is associated with elevated ASD symptoms. This is the first study in a large cohort that robustly accounts for the phenotypic overlap between the ADHD and ASD. Equivocal findings in previous volumetric studies of ADHD and ASD may be due to the fact that issues of comorbidity have generally been ignored in studies to date. Assessing the range of volumetric profiles associated with ADHD coupled with mild ASD symptoms, through to ADHD coupled with severe ASD symptoms, will help to develop a more nuanced understanding of the pathophysiology associated with the continuous distribution and overlap of these two disorders.

## Supporting Information

Table S1 **Medication Information.**
(DOCX)

Table S2 **Distribution of Scanning over Two Sites.**
(DOCX)

Table S3 **Correlation between ASD score and ADHD symptom levels.**
(DOCX)

Table S4 **Final, generalised mixed-effect model for ASD score modelled against Grey Matter** **Residual and White Matter Volumes.**
(DOCX)

Table S5 **Final, generalised mixed-effect model for ASD score modelled against Grey Matter and White Matter Volumes using only participants with an ASD score of 20 or higher.**
(DOCX)

Diagnostic Algorithm S1 **Diagnostic algorithm for ADHD in the NeuroIMAGE sample.**
(DOCX)

Questionnaire Items S1 **Children’s Social and Behavioural Questionnaire (CSBQ) Summary.**
(DOCX)
