# Supplementary material for: Brain Volumetric Correlates of Autism Spectrum Disorder Symptoms in Attention Deficit/Hyperactivity Disorder
Source: PLoS One. 2014 Jun 30;9(6):e101130. doi: 10.1371/journal.pone.0101130 (PMC4076257; doi:10.1371/journal.pone.0101130)
Supplement: Table S1 — Medication Information. (DOCX) [file pone.0101130.s001.docx]

|  | ADHD |
| --- | --- |
|  | n=180 |
| Atomoxetine (Straterra) | 10 |
| Dexamphetamine sulphate | 2 |
| Methylphenidate - immediate release (Ritalin) | 26 |
| Methylphenidate - extended release (Concerta) | 48 |
| Methylphenidate - extended release (Equasym) | 2 |
| Methylphenidate - extended release (Medikinet) | 5 |
| Dexamphetamin sulphate and Concerta | 2 |
| Ritalin and Concerta | 13 |
| Concerta and Medikinet | 1 |
| No ADHD Medication | 71 |
| Mean age of ADHD medication group (n=109) | 15 |
| Mean age of ADHD non-medication group (n=71) | 18 |

The trade names of the ADHD medications are noted in brackets.
