# Supplementary material for: Brain Volumetric Correlates of Autism Spectrum Disorder Symptoms in Attention Deficit/Hyperactivity Disorder
Source: PLoS One. 2014 Jun 30;9(6):e101130. doi: 10.1371/journal.pone.0101130 (PMC4076257; doi:10.1371/journal.pone.0101130)
Supplement: Table S2 — Distribution of Scanning over Two Sites. (DOCX) [file pone.0101130.s002.docx]

|  | Avanto | Sonata |
| --- | --- | --- |
| Control | 70 | 76 |
| Unaffected Siblings | 81 | 37 |
| ADHD | 152 | 28 |
