# Supplementary material for: Brain Volumetric Correlates of Autism Spectrum Disorder Symptoms in Attention Deficit/Hyperactivity Disorder
Source: PLoS One. 2014 Jun 30;9(6):e101130. doi: 10.1371/journal.pone.0101130 (PMC4076257; doi:10.1371/journal.pone.0101130)
Supplement: Table S3 — Correlation between ASD score and ADHD symptom levels. (DOCX) [file pone.0101130.s003.docx]

|  | r | t-value | p-value |
| --- | --- | --- | --- |
| Con | 0.165 | 2.011 | 0.046 |
| Unaffected siblings | 0.043 | 0.465 | 0.643 |
| ADHD | 0.254 | 3.507 | 0.001 |
