# Supplementary material for: Brain Volumetric Correlates of Autism Spectrum Disorder Symptoms in Attention Deficit/Hyperactivity Disorder
Source: PLoS One. 2014 Jun 30;9(6):e101130. doi: 10.1371/journal.pone.0101130 (PMC4076257; doi:10.1371/journal.pone.0101130)
Supplement: Table S5 — Final, generalised mixed-effect model for ASD score modelled against Grey Matter and White Matter Volumes using only participants with an ASD score of 20 or higher. (DOCX) [file pone.0101130.s005.docx]

Fixed Effects:

|  | Estimate | Standard Error | t-value | p-value |
| --- | --- | --- | --- | --- |
| WM resid | -1.340 | 0.430 | 3.114 | 0.0032 |
| GM | -0.0155 | 0.0318 | -0.488 | 0.628 |
| Age | -0.102 | 0.382 | -0.266 | 0.791 |
| Male | 0.970 | 1.654 | 0.587 | 0.560 |
| WM resid x GM | -0.003 | 0.000 | -3.149 | 0.0029 |
|  |  |  |  |  |
|  |  |  |  |  |

Random Effects:

|  | Variance | S.D. |
| --- | --- | --- |
| Family Relatedness | 23.08 | 4.804 |
| IQ | 0.000 | 0.000 |
| Total ADHD Score | 0.000 | 0.000 |
| ADHD medication | 1.448 | 1.203 |
| Scanner Type | 0.000 | 0.000 |

Formula:

$$\begin{matrix} ASD\text{ }symptoms\text{ }\sim GM\text{ }*\text{ }WM\text{ }resid\text{ }+\text{ }Diagnosis\text{ }*\text{ }Age\text{ }+\text{ }Gender\text{ }+ \\ \left(1|Total\text{ }ADHD\text{ }symptoms\right)\text{ }+ \\ \left(1|\text{ }Family\right) + \\ \left(1|\text{ }IQ\right)\text{ }+ \\ \left(1|ADHD\text{ }medication\right)\text{ }+ \\ \left(1|\text{ }Scanner\text{ }type\right) \end{matrix}$$

"~" means modelled against, and "(1| factor)" means that a factor is included as a random effect.

A generalised mixed-effect model is run using normalised volumes of grey and white matter as explanatory variables together with age as a random effect. ASD score is set as the response variable. The final model is derived following an iterative model selection procedure that involves comparing successive models using Akaike’s Information Criterion (see Methods for detailed description of model selection procedure). ASD score refers to an aggregate score from the four Children's Social and Behavioural Questionnaire (CSBQ) subscales, (1) social interest, (2) social understanding, (3) stereotypy and (4) resistance.

Abbreviations: WM, normalised WM volume; GM, normalised GM volume; US, unaffected siblings; WM:GM, WM by GM interaction; US: Age, unaffected sibling by Age interaction; ADHD:Age, ADHD by Age interaction.
