# Supplementary material for: Brain Volumetric Correlates of Autism Spectrum Disorder Symptoms in Attention Deficit/Hyperactivity Disorder
Source: PLoS One. 2014 Jun 30;9(6):e101130. doi: 10.1371/journal.pone.0101130 (PMC4076257; doi:10.1371/journal.pone.0101130)
Supplement: Questionnaire Items S1 — Children’s Social and Behavioural Questionnaire (CSBQ) Summary. (DOCX) [file pone.0101130.s007.docx]

The CSBQ consists of the following questions that are divided into 6 subscales as noted below.

*Not Tuned:*

Gets angry quickly

Does not know when to stop

Extremely stubborn

Stays angry for a long time e.g., when he/she does not get his/her

Is disobedient

Draws excessive attention to him/herself

Shows sudden changes of mood

Makes a fuss over little things; ‘‘makes a mountain of a mole-hill’’

Over-reacts to everything and everyone

Cannot be corrected in situations in which he/she has done

Makes inconsiderate remarks e.g., remarks that are painful to others

*Reduced Social Interest:*

Has little or no need for contact with others

Makes little eye contact

Does not seek comfort when he/she is hurt or upset

Dislikes physical contact e.g., does not want to be touched or hugged

Does not respond to initiatives by others e.g., does not play along when asked

Does not initiate play with other children

Acts as if others are not there

Lives in a world of his/her own

Does not show his/her feelings in facial expressions and/or bodily posture

Does not look up when spoken to

Cannot be made enthusiastic about anything

Does not appreciate it when someone else is hurt or sad

*Orientation Problems:*

Does things without realizing the aim e.g. has to be reminded to finish things

Has no sense of time

Takes in information with difficulty

Has difficulties doing two things simultaneously

Does not appreciate danger

Gets lost easily e.g., when out with someone

Barely distinguishes between strangers and familiar people

*Reduced Social Understanding:*

Takes things literally e.g., does not understand certain expressions

Does not understand jokes

Does not fully understand what is being said to him/her

Is exceptionally naive; believes anything you say

Frequently says things that are not relevant to the conversation

Talks confusedly; jumps from one subject to another in speaking

Only talks about things that are of concern for himself/herself

*Stereotyped Behaviour:*

Constantly feels objects

Smells objects

Makes odd, fast movements with fingers or hands

Is extremely pleased by certain movements and keeps doing them

Flaps arms/hands when excited

Is fascinated by certain colors, forms, or moving objects

Sways to and fro

Is unusually sensitive to certain sounds

*Resistance to change:*

Remains clammed up in new situations or if change occurs

Panics in new situations or if change occurs

Opposes change
